# The bone–muscle connection in breast cancer: implications and therapeutic strategies to preserve musculoskeletal health

**DOI:** 10.1186/s13058-022-01576-2

**Published:** 2022-11-23

**Authors:** Tarah J. Ballinger, William R. Thompson, Theresa A. Guise

**Affiliations:** 1 Department of Medicine, Indiana University School of Medicine, 535 Barnhill Dr. RT 473, Indianapolis, IN 46202 USA; 2 Department of Endocrine Neoplasia and Hormonal Disorders, MD Anderson Cancer Center, Houston, TX USA

**Keywords:** Sarcopenia, Bone remodeling, Breast cancer, Cytokines, Vibration

## Abstract

Breast cancer and its therapies frequently result in significant musculoskeletal morbidity. Skeletal complications include bone metastases, pain, bone loss, osteoporosis, and fracture. In addition, muscle loss or weakness occurring in both the metastatic and curative setting is becoming increasingly recognized as systemic complications of disease and treatment, impacting quality of life, responsiveness to therapy, and survival. While the anatomical relationship between bone and muscle is well established, emerging research has led to new insights into the biochemical and molecular crosstalk between the skeletal and muscular systems. Here, we review the importance of both skeletal and muscular health in breast cancer, the significance of crosstalk between bone and muscle, and the influence of mechanical signals on this relationship. Therapeutic exploitation of signaling between bone and muscle has great potential to prevent the full spectrum of musculoskeletal complications across the continuum of breast cancer.

## Background

Musculoskeletal complications are a major clinical challenge in the management of early- and late-stage breast cancer. The majority of breast cancers are treated with estrogen deprivation therapy, which increases bone turnover, resulting in increased rates of bone loss and osteoporosis. Beyond this, breast cancer has a high propensity to metastasize to bone. Emerging evidence shows states of high bone turnover caused by estrogen deprivation and/or bone metastases alter the bone microenvironment to accelerate bone loss and fuel tumor cell progression, resulting in a damaging feed-forward cycle.

Beyond the skeletal complications of breast cancer, tumor progression, chemotherapy, and estrogen deprivation can result in muscle loss and muscle dysfunction. Given the importance of physical function and physical activity to quality of life and survival in breast cancer, this has significant potential clinical implications. Skeletal and muscle loss are interrelated both anatomically and biochemically. Understanding of the importance of both tissue systems and their relationship can unlock novel therapeutic strategies for managing the full spectrum of musculoskeletal complications in breast cancer.

## High bone turnover effects skeletal health and promotes in-bone tumor growth

The majority of breast cancers are estrogen receptor (ER) positive. Five to ten years of complete estrogen deprivation with aromatase inhibitor (AI) therapy is standard of care in the curative setting, significantly reducing recurrence rates [1]. The effectiveness of AI therapy is complicated by musculoskeletal toxicity, including pain, weakness, and bone loss. Beyond an impact on quality of life, toxicity results in the majority of women not taking AIs as prescribed [2]. Non-compliance is associated with significantly worse disease-free survival and is an independent predictor of increased mortality [3].

Unlike selective estrogen receptor modulators (SERMS) such as tamoxifen, aromatase inhibition results in increased bone turnover, accelerated bone loss, and increased fracture risk. In the ATAC trial comparing the AI anastrozole to tamoxifen in the adjuvant setting, markers of bone resorption (C-telopeptide, N-telopeptide) and bone formation (alkaline phosphatase, procollagen type-1N-propeptide) were significantly increased in patients receiving anastrozole [4]. Bone mineral density (BMD) at 5 years increased in patients receiving tamoxifen, while lumbar spine BMD decreased by 6.1% in the AI group [5]. The mechanism of AI-induced bone loss is not completely understood, but is likely due to rapid depletion of peripheral estrogen synthesis through inhibition of the rate limiting step of estrogen biosynthesis from testosterone in adipose tissue [6]. Estrogen directly regulates bone destroying osteoclasts and bone building osteoblasts by stimulating anti-resorptive factors, reducing RANK ligand signaling to decrease osteoclast activation [7]. Additionally, estrogen decreases T cell production of inflammatory cytokines that activate osteoclasts; therefore, in the absence of estrogen, there is an increase in tumor necrosis factor (TNF)-alpha, IL-1 and IL-6, stimulating bone loss [8, 9].

In addition to the detrimental effects of anti-estrogen therapy on bone health, preclinical data suggests that the increased bone turnover caused by AI therapy may prime the bone microenvironment to facilitate breast cancer growth in bone. Accelerated bone destruction, such as in states of estrogen deprivation, causes release and activation of growth factors stored in the mineralized bone matrix, including insulin-like growth factor, fibroblast growth factors, platelet-derived growth factor, and TGFβ. These factors increase production of osteolytic factors, stimulating tumor growth and invasive behavior in an already highly vascularized area [10]. In mouse models of breast cancer bone metastases established by injection of human MDA-MB-231 breast cancer cells, expression of osteolytic factors (RANKL, Cathepsin-K, and MMP9) was higher in ovariectomized (OVX) mice mimicking the postmenopausal bone microenvironment, compared with sham operation. Tumor growth significantly greater in the OVX mice only in bone, while estrogen status had no impact on tumor growth outside of bone. Furthermore, suppression of bone turnover using the bisphosphonate zoledronic acid prevented tumor growth in bone in the postmenopausal model without impact on tumor sites outside of bone [11]. Similarly, in a model of OVX mice treated with the AI letrozole and inoculated with MDA-MB-231 triple negative breast cancer cells (chosen to study the bone microenvironment without the effect of direct inhibition of tumor ER signaling), osteolytic bone metastases were increased in AI-treated mice, compared to both sham-treated mice and OVX mice without AI. Zoledronic acid reduced osteoclastogenesis and tumor burden in bone of the estrogen-deprived, AI-treated mice [12]. Taken together, these data support the hypothesis that increased bone turnover in estrogen deprivation states fuel metastatic tumor growth in bone.

The effect of estrogen deprivation to increase bone destruction and enhance metastatic potential within the bone microenvironment is further supported by clinical data demonstrating improved disease-free survival with the use of anti-resorptive agents in the adjuvant setting. This benefit is seen only in postmenopausal, but not pre-menopausal, women [13]. Both bisphosphonates and the RANK ligand inhibitor denosumab have been shown to prevent BMD loss, and reduction in bone turnover has been associated with improvements in disease-free survival [14, 15]. In the Early Breast Cancer Trialists Cooperative Group meta-analysis of adjuvant bisphosphonate therapy in breast cancer patients, disease recurrence was reduced only in estrogen-deprived women and was most significant for reductions in bone metastases (any recurrence RR 0.86, 95% CI 0.78–0.94, *p* = 0.002, bone recurrence RR 0.72, 95% CI 0.60–0.86, *p* = 0.0002) [14]. Furthermore, high bone turnover states appear prognostic of in-bone breast cancer recurrences, as well as disease progression and death. Evaluation of baseline bone turnover markers (P1NP, CTX, and 1-CTP) in the randomized AZURE study of adjuvant zoledronic acid demonstrated a clear association between bone turnover and later development of recurrence in bone, but not metastases outside bone. These bone metastases events occurred later than the first two years of follow-up, indicating higher bone turnover was not simply a marker of undetected, already present bone metastases [16]. This could indicate an enriched bone microenvironment that may activate dormant cancer cells in bone.

## Muscle health impacts physical functioning and breast cancer outcomes

In addition to detrimental effects on skeletal health and the bone microenvironment, breast cancer and its treatment regimens impact structure and function of the muscular system. While several studies have investigated the impact of body weight in breast cancer, fewer have investigated the impact of true body composition and muscle (Table 1). Bone loss at menopause is associated with loss of muscle mass and function, likely related both to direct effects of ER signaling on muscle tissue and a decline in physical activity. In addition, hormone replacement therapy after menopause attenuates muscle loss and improves myogenic response to exercise [17, 18]. Therefore, it is hypothesized that anti-estrogen therapy in breast cancer may further muscle loss and dysfunction that already occurs with menopause and aging. Preclinical models of mice undergoing OVX found a similar reduction in lean mass with the AI letrozole treatment compared to placebo, but a significant reduction in muscle specific force of the extensor digitorum longus muscle in those mice treated with both OVX and the AI (*p* < 0.05) [12]. In clinical populations, the impact of endocrine therapy on muscle mass is inconsistent, and its effect on true muscle function is underexplored. Our prior work evaluating body composition and energetic capacity of stage I-III breast cancer patients at baseline and following primary therapy found a significant reduction in muscle power by 6 months of endocrine therapy, including those receiving AIs, which occurred without a change in lean mass. A similar effect was seen in patients who received chemotherapy, while no significant change in function was seen in those patients who did not receive systemic therapy [19].

**Table 1:** Summary of studies evaluating the impact of muscle on breast cancer outcomes

Author, year	Population	Measures	Outcome
Sheehan et al. [20]	Retrospective Stage IV, ER negative *n* = 152	CT scan Sarcopenia (SMI ≤ 41 cm^2^/m^2^) Low SMD (< 41 HU, < 33 HU if BMI ≥ 25)	Sarcopenia at diagnosis not associated with OS. Low SMD reduced 2 year OS, HR 1.72 (1.09–2.72)
Caan et al. [21]	Retrospective Stage II, III *n* = 3241	CT scan Sarcopenia (SMI < 40 cm^2^/m^2^) Low SMD (< 37.8 HU)	Sarcopenia associated with OS, HR 1.41 (95% CI 1.18–1.69) SMD not associated with OS
Deluche et al. [22]	Retrospective Stage I–III *n* = 119	CT scan Sarcopenia (SMI < 41 cm^2^/m^2^)	Absence of sarcopenia associated with better DFS (HR 0.3, 95% CI 0.1–0.8, *p* = 0.02) and OS (HR 0.3, 95% CI 0.1–0.99, *p* = 0.05)
Shachar et al. [23]	Retrospective Stage IV, taxane chemotherapy *n* = 40	CT scan Sarcopenia (SMI < 41 cm^2^/m^2^)	Sarcopenia associated with higher grade 3–4 toxicity (18% vs. 57%, *p* = 0.02). No association with survival
Rier et al. [24]	Retrospective Stage IV, first line chemotherapy *n* = 166	CT scan Sarcopenia (SMI < 41 cm^2^/m^2^) Low SMD (< 41 HU, < 33 HU if BMI ≥ 25)	Low SMD associated with lower OS (HR 2.04, 95% CI 1.34–3.12, *p* = 0.001). SMI had no association with OS
Villasenor et al. [25]	Prospective Stage 0–III *n* = 471	DEXA scan Sarcopenia (SMI < 5.45 kg/m^2^)	Sarcopenia associated with OS (HR 2.86, 95% CI 1.67–4.89)
Prado et al. [26]	Retrospective Stage IV, capecitabine chemotherapy *n* = 55	CT scan Sarcopenia (SMI < 38.5 cm^2^/m^2^) Low SMD (< 41 HU, < 33 HU if BMI ≥ 25)	Sarcopenia associated with grade 3–4 toxicity (20% vs. 50%, *p* = 0.03) and shorter TTP (173 vs 101 days, *p* = 0.05)

*CT* computed tomography, *SMI* skeletal muscle index, *SMD* skeletal muscle density, *HU* Hounsfield units, *OS* overall survival, *HR* hazard ratio, *DFS* disease free survival, *TTP* time to progression

Chemotherapy is known to have direct effects on muscle, including both loss of lean mass and reduction in contractile function of muscle tissue. Several observational clinical studies have shown worse physical function following chemotherapy contributing to the syndrome of cancer-related fatigue, but the muscle-specific mechanisms remain unclear. Preclinical work suggests cytotoxic therapy induces protein degradation in muscle via high oxidative stress and activation of the NF-κB pathway and induction of the ubiquitin–proteasome system [27]; likewise, chemotherapy-induced activation of mammalian target of rapamycin complex 1 (mTORC1) in muscle reduces protein synthesis, contributing to atrophy [28]. Recent breast cancer-specific work evaluating the effect of carboplatin on mice with MDA-MB-231 bone metastases found that carboplatin alone induced loss of both bone and lean muscle mass, regardless of tumor response to treatment. Further extension of this work found that inhibition of bone turnover with zoledronic acid in the presence of carboplatin did not prevent muscle atrophy but did preserve bone mass and muscle strength [29]. This further supports potential for muscle specific benefits of anti-resorptive therapy on muscle function in cancer patients.

Loss of muscle mass and/or loss of muscle function has significant clinical implications. This is often referred to as sarcopenia, a term which may not reflect the most specific pathophysiology as muscle dysfunction can occur without the loss of muscle mass and in the absence of cachexia. In patients with advanced cancer, sarcopenia (defined as low muscle mass on computed tomography (CT) scan) is associated with significantly worse overall and cancer-specific survival (*n* = 7843, OS HR 1.44, 95% CI 1.32–1.56, *p* < 0.001, CSS HR 1.93, 95% CI 1.38–2.70, *p* < 0.001) [30]. Studies to date evaluating this relationship specifically in metastatic breast cancer are small and inconclusive, and few evaluate actual muscle function (Table 1). While loss of muscle mass is traditionally thought to be associated with increased morbidity and mortality in late-stage disease, the existing literature also supports a significant impact on prognosis in early-stage breast cancer. In a large study by Caan et al. estimating muscle mass on computed tomography (CT) scans obtained in 3282 patients with stage II-III breast cancer, 34% of patients had sarcopenia (defined as skeletal muscle index < 40 cm^2^/m^2^). Patients with sarcopenia at the time of diagnosis had a significantly higher risk of death (HR 1.41, 95% CI 1.18–1.69), compared to those who were not sarcopenic [21]. Notably, this association was stronger than that observed for body mass index (BMI), a measure only reflecting body weight and not accounting for true body composition; this may have misclassified women with breast cancer at risk for poor outcome in prior studies and may explain discrepant data on the impact of weight in breast cancer.

Beyond muscle mass, the quality of muscle is a significant outcome to consider. Myosteatosis or fatty infiltration into muscle is inversely proportional to the density of muscle and is also an independent predictor of chemotherapy toxicity and worse survival in several populations of individuals with both early- and late-stage cancer [23, 31]. In addition, muscle density is a better predictor of poor muscle function than muscle mass in both geriatric and oncology populations [32]. In an analysis of muscle mass and muscle density on CT scans of older patients with cancer in the Carolina Senior Registry, skeletal muscle mass was not associated with physical function impairments, while muscle quality was significantly associated with limitations in activities of daily living, climbing, and walking, and an increased score on the Timed Up and Go test [32]. Impact of true muscle function is paramount to quality of life and physical independence and is a significant independent predictor of mortality in the general population [33, 34]. In a prospective study of older women with breast cancer, self-reported decline in physical function was predictive for shorter 10-year overall survival (HR 1.34, 95% CI 1.1–1.6) [35]. Given that the majority of breast cancer patients are older adults, detriments to the muscular system are significant, exacerbating the decline that already occurs with aging and deconditioning.

## Cytokine-mediated crosstalk between bone and muscle

Historically, the relationship between bone and muscle has been thought of as primarily anatomical. Bone provides attachment points to support muscle structure and movement, while muscle activity generates anabolic strain to the bone microarchitecture. The retrospective and prospective literature supports the coexistence and linear relationship of bone loss (osteoporosis) and muscle loss (sarcopenia). Aging and disuse result in loss of both skeletal and muscle tissue, while physical activity maintains the strength of both systems. In aging and several chronic conditions, lower bone density and lean muscle mass have been observed to be interrelated and coexistent [36], supporting the concept of a cohesive musculoskeletal system. In an analysis of the Osteoporosis Fracture Prevention study of postmenopausal women, those with sarcopenia according to DEXA, grip strength, and walking speed had a twelve-fold higher odds of osteoporosis (OR 12.9, 95% CI 3.1–53.5), unaffected by age, body mass index, physical activity levels, or hormone replacement therapy [37]. Historically, bone and muscle endpoints have been studied in isolation and a causative relationship was difficult to establish until recently.

Independent, and complementary to, mechanical and anatomic connections, muscle and bone are both active endocrine organs communicating by autocrine and paracrine signaling. Bone is a storehouse for growth factors and cytokines that are released into circulation during states of increased bone destruction. Late osteoblasts release osteocalcin, a hormone peptide that increases insulin sensitivity and mitochondrial content in skeletal muscle [38]. Osteoblasts also produce insulin-like growth factor (IGF1) and bone morphogenetic protein 2 (BMP2) during states of active bone resorption. IGF1 signaling activates Akt in skeletal muscle, resulting in muscle hypertrophy and increased muscle force generation [39]. BMP2 promotes hypertrophy and resistance to atrophy in skeletal muscle [40]. While these factors result in a hypertrophic response in muscle, several bone released cytokines are associated with reduced function of muscle. Members of the TGFβ superfamily, myostatin, activin, and TGFβ, are produced by osteoblasts, deposited into the mineralized bone matrix, and released during osteoclastic bone resorption. In mouse models of increased circulating activin, there is a significant dose-dependent loss of skeletal muscle and subsequent loss of function mediated by increased transcription of ubiquitin ligases, decreased Akt signaling, and increased muscle fibrosis. In contrast to loss of mass, TGFβ induces significant loss of muscle strength [41].

**Fig. 1:**
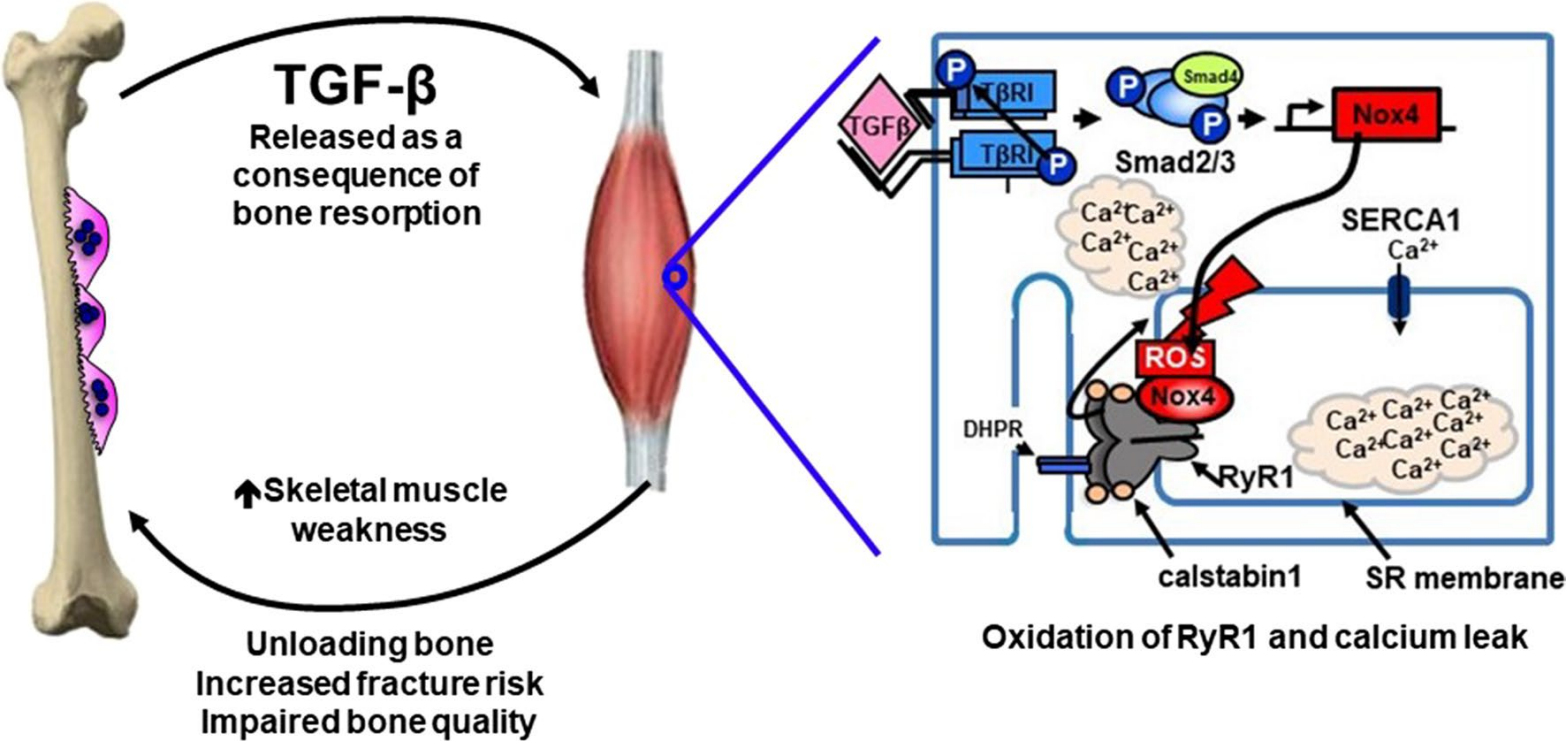
States of high bone turnover release TGF-β into systemic circulation. In skeletal muscle, bone-derived TGF-β results in upregulation of NADPH oxidase 4 (Nox4), producing reactive oxygen species that destabilize RyR1-calstabin1 complex. This leads to calcium leak and muscle weakness, further unloading and weakening bone strength in a feed-forward cycle of musculoskeletal decline (figure adapted and modified from Waning and Guise et al., *Nature Med,* 2015)

TGFβ in particular has significant effects in the bone microenvironment to increase osteoclastic bone resorption and decrease osteoblast differentiation, facilitating tumor growth in bone. Beyond this, novel work has elucidated a mechanism by which TGFβ exerts systemic effects on muscle outside of the immediate bone microenvironment. In mouse models of osteolytic breast cancer bone metastases, significant muscle weakness was seen that was not present in mice with primary breast cancer and no bone metastases [42]. Importantly, this weakness was proportional to tumor burden in bone and occurred independent of loss of muscle mass. Weakness was systemic, not restricted to the limb or area of bone metastases, and was not associated with reduced food consumption. Similar muscle dysfunction was also noted in other osteolytic tumor models of lung and prostate cancer, as well as multiple myeloma [42]. Mice in these models had reduced grip strength and muscle contractility, measured by muscle specific force of the extensor digitorum longus muscle. Mechanistic work determined that TGFβ-mediated SMAD3 phosphorylation of NADPH oxidase 4 (Nox4) in skeletal muscle results in generation of reactive oxygen species and oxidation of the ryanodine receptor calcium release channel (RyR1) (Fig. 1). RyR1 is present in the sarcoplasmic reticulum of skeletal muscle and functions to regulate calcium release in normal muscle contraction. When oxidized, the receptor loses its stabilizing protein, calstabin1, and becomes “leaky”. This results in lower tetanic calcium and weakens muscle force. In mouse models of osteolytic breast cancer, inhibition of TGFβ activity, TGFβ release from bone (using zoledronic acid), Nox4, or RyR1 calcium leak all restored muscle force production [42]. This suggests a systemic mechanism for weakness associated with breast cancer bone metastases, indicates muscle dysfunction that occurs independent of frank muscle loss, and suggests potential targets to improve muscle weakness associated with bone destruction.

The hypothesis that reducing cancer-induced bone turnover may improve muscle function has yet to be tested in clinical populations. Of interest, the ZAP trial, a single arm, phase II, prospective trial of zoledronic acid in women receiving adjuvant AIs, compared rates of aromatase inhibitor-induced musculoskeletal syndrome (AIMSS) to historical rates [43]. After 6 months of zoledronic acid, participants reported significantly less functional disability by the Health Assessment Questionnaire Disability Index (HAQ-DI) compared to the historical controls who had not received a bisphosphonate (OR 0.32, 95% CI 0.16–0.64, *p* = 0.003). It is not clear whether this functional questionnaire reflects true muscle function. These results are hypothesis generating and support efforts to translate preclinical work and determine the impact of skeletal protective therapy on muscle function in prospective clinical trials.

## Exercise and mechanical signals to preserve both skeletal and muscular health

Aerobic and/or resistance training exercise is a very well established, effective therapeutic strategy to maintain the integrity of both bone and muscle exercise can improve bone density loss in postmenopausal women, and a randomized trial in breast cancer survivors found significant improvement in markers of bone formation, including osteocalcin, with exercise compared to usual care [44]. The ability of exercise to be anabolic to the musculoskeletal system is multifactorial but stems primarily from the influence of mechanical signals translated into biochemical signals at the tissue and cellular level. Bone, muscle, and adipose cells originate from a common mesenchymal precursor. These mesenchymal stem cells (MSCs) are positioned within the bone marrow and are sensitive to various forces including fluid shear stress, strain of the cell membrane from micro-deformations of bone, forces from muscle contraction, and oscillatory/vibrational signals induced by exercise [45]. Mechanical strain recruits intracellular signals to focal adhesion complexes resulting in increased connection of the extracellular matrix with the actin cytoskeleton. Such connections enable both translocation of mechanically sensitive signaling molecules and direct transmission of force from the plasma membrane to the nucleus. Sensation of mechanical signals by the nucleus in MSCs regulates gene expression to effect both stem cell fate and regulation of cell shape and structure [46]. Mechanical signals increase expression of osteogenic genes such as *RUNX2* and *SOX9* via increases in the WNT effector protein β-catenin, while downregulating expression of *PPAR-γ,* a driver of adipogenesis [47, 48]. In contrast, periods of inactivity result in decreased differentiation of MSCs into osteoblasts, with increases in *PPAR-γ* and adiponectin, as well as RANKL signaling increasing osteoclastic bone resorption [49]. Thus, exercise can alter the fate of bone marrow MSCs based upon mechanical qualities sensed by both the extracellular matrix and internal cytoskeleton.

Despite the known benefits of exercise, physical activity decreases after breast cancer diagnosis and treatment, exacerbating insult to the musculoskeletal system. Reduced physical function and weakness increase risk of falls and pathologic fracture, while frailty and musculoskeletal pain further compromise the safety and capability to effectively participate in exercise. The majority of breast cancer survivors do not meet physical activity guidelines [25], and many physical activity interventions suffer from a lack of enrollment of participants who are most deconditioned and in need. Preclinical evidence suggests alternative mechanisms of mechanical stimulation, such as low intensity vibration (LIV), can simulate exercise and be anabolic to both bone and muscle, particularly in those who cannot or will not exercise.

While typical exercise regimens (such as running) deliver mechanical signals through high impact strain (> 1000µɛ) in few repetitions, lower strain signals (< 100µɛ) delivered over many repetitions may have a similar benefit. LIV is a high-frequency (30 Hz), low-magnitude (< 1* g*) mechanical signal that simulates the vibratory twitch of type II muscle fibers and recapitulates the mechanical stimulation experienced during exercise [50]. In preclinical work, LIV activates cellular mechanosensitive pathways and alters gene expression, reducing adipogenic *PPARγ* expression, while upregulating osteogenic genes *RUNX2* and *SOX9* [46, 51]. Specifically, in mice mimicking a state of estrogen deficiency, immediate LIV after OVX slowed accumulation of adipose tissue in bone marrow and protected loss of trabecular bone [52]. Importantly, LIV in OVX mice also preserved the muscle satellite cell population that is critical to muscle health and repair, and has significant implications for physical function [51]. In clinical populations, the optimal dose and time period of LIV remain unclear and depend on which musculoskeletal endpoint is studied. LIV has been found to improve cortical and trabecular bone mass in young women with low bone density [53], balance and mobility in osteopenic postmenopausal women [54], and bone mineral density in childhood cancer survivors [55]. We are currently evaluating the impact of LIV on bone quality, muscle function, and osteolytic cancer burden in mouse models of breast cancer bone metastases. In these studies, mice treated with complete estrogen deprivation (OVX and AI) and LIV had improved trabecular bone mass due to reduced osteoclast and increased osteoblast activity, improved muscle strength, and reduced body fat [56]. In addition, our ongoing randomized clinical trial will evaluate the efficacy of LIV to preserve muscle strength, physical function, and bone mass in women beginning AIs who do not currently exercise (NCT03712813).

Future work will also evaluate the lesser-known impact of mechanical signals on cancer cells themselves. In mouse models of ovarian cancer [57] and multiple myeloma [58], delivery of exogenous mechanical signals in the form of LIV was associated with reduced cancer induced bone loss, as well as reduced tumor burden; this raises the question of whether beneficial effects of mechanical signals are secondary to effects on the musculoskeletal system or due to direct regulation of cancer cells. Ongoing work from our group is using in vitro culture models to examine how mechanical signals direct biochemical and biophysical changes in breast cancer cells and alter indirect paracrine signaling to bone cells. In these studies, application of LIV results in a threefold reduction in MDA-MB-231 cell invasion through the extracellular matrix, likely mediated by decreased expression of matrix metalloproteinases that are required to degrade the matrix. Furthermore, the compliance (i.e., cell stiffness) of cancer cells is inversely proportional to metastatic potential. In our work, LIV treatment significantly increased plasma membrane stiffness of MDA-MB-2321 cells, measured by atomic force microscopy [59]. We also found that disruption of the Nucleoskeleton and Cytoskeleton (LINC) complex in MDA-MB-231 cells exposed to LIV abrogates the beneficial effects of the mechanical stimulus. Following knockdown of Sun proteins within the LINC complex, LIV signals no longer reduced invasion, decreased production of osteolytic factors, or enhanced cytoskeletal remodeling in tumor cells. Additionally, LIV increased expression of LINC complex proteins, suggesting that mechanical signals enhance nuclear-cytoskeletal connectivity, making cells more sensitive to mechanical signaling. Recent work has found decreased expression of LINC proteins in breast cancer [60]; therefore, loss of the LINC complex may increase invasive pathogenesis, an effect that may be offset by the introduction of mechanical signals.

## Conclusion

Breast cancer and cancer treatment, most notably estrogen deprivation therapy, result in significant and well-characterized long-term skeletal complications, including osteoporosis and fracture. However, the less explored “musculo” part of musculoskeletal health is critical as loss of muscle mass and function more immediately effect physical function, exercise capacity, and quality of life, ultimately impacting survival outcomes. Increased bone destruction associated with estrogen deprivation (and possibly chemo- and radiation) therapy or bone metastases has extensive complications beyond skeletal-related events, contributing to further progression of cancer in bone and to muscle wasting and dysfunction. Emerging evidence indicates that transduction of mechanical signals can reduce bone turnover and influence mesenchymal and satellite cells to improve the health of both bone and muscle, while reducing adiposity. Future research will determine the direct impact of mechanical signals on the tumor microenvironment and tumor metastatic potential and work to determine the optimal therapeutic combination of pharmacologic and non-pharmacologic strategies to diminish the burden of musculoskeletal morbidity in breast cancer.

## Data Availability

Not applicable.

## References

[1] Goss PE, Ingle JN, Pritchard KI, Robert NJ, Muss H, Gralow J (2016). Extending aromatase-inhibitor adjuvant therapy to 10 years. N Engl J Med.

[2] Murphy CC, Bartholomew LK, Carpentier MY, Bluethmann SM, Vernon SW (2012). Adherence to adjuvant hormonal therapy among breast cancer survivors in clinical practice: a systematic review. Breast Cancer Res Treat.

[3] Chirgwin JH, Giobbie-Hurder A, Coates AS, Price KN, Ejlertsen B, Debled M (2016). Treatment adherence and its impact on disease-free survival in the breast international group 1–98 trial of tamoxifen and letrozole, alone and in sequence. J Clin Oncol.

[4] Eastell R, Hannon RA, Cuzick J, Dowsett M, Clack G, Adams JE (2006). Effect of an aromatase inhibitor on bmd and bone turnover markers: 2-year results of the Anastrozole, Tamoxifen, Alone or in Combination (ATAC) trial (18233230). J Bone Miner Res.

[5] Eastell R, Adams JE, Coleman RE, Howell A, Hannon RA, Cuzick J (2008). Effect of anastrozole on bone mineral density: 5-year results from the anastrozole, tamoxifen, alone or in combination trial 18233230. J Clin Oncol.

[6] Geisler J, Haynes B, Anker G, Dowsett M, Lonning PE (2002). Influence of letrozole and anastrozole on total body aromatization and plasma estrogen levels in postmenopausal breast cancer patients evaluated in a randomized, cross-over study. J Clin Oncol.

[7] Frenkel B, Hong A, Baniwal SK, Coetzee GA, Ohlsson C, Khalid O (2010). Regulation of adult bone turnover by sex steroids. J Cell Physiol.

[8] Jilka RL, Hangoc G, Girasole G, Passeri G, Williams DC, Abrams JS (1992). Increased osteoclast development after estrogen loss: mediation by interleukin-6. Science.

[9] Abitbol A, Rabasa-Lhoret R, Messier V, Legault L, Smaoui M, Cohen N (2018). Overnight glucose control with dual- and single-hormone artificial pancreas in type 1 diabetes with hypoglycemia unawareness: a randomized controlled trial. Diabetes Technol Ther.

[10] Buijs JT, Stayrook KR, Guise TA (2011). TGF-beta in the bone microenvironment: role in breast cancer metastases. Cancer Microenviron.

[11] Ottewell PD, Wang N, Brown HK, Reeves KJ, Fowles CA, Croucher PI (2014). Zoledronic acid has differential antitumor activity in the pre- and postmenopausal bone microenvironment in vivo. Clin Cancer Res.

[12] Wright LE, Harhash AA, Kozlow WM, Waning DL, Regan JN, She Y (2017). Aromatase inhibitor-induced bone loss increases the progression of estrogen receptor-negative breast cancer in bone and exacerbates muscle weakness in vivo. Oncotarget.

[13] Coleman RE, Collinson M, Gregory W, Marshall H, Bell R, Dodwell D (2018). Benefits and risks of adjuvant treatment with zoledronic acid in stage II/III breast cancer. 10 years follow-up of the AZURE randomized clinical trial (BIG 01/04). J Bone Oncol..

[14] Early Breast Cancer Trialists' Collaborative G (2015). Adjuvant bisphosphonate treatment in early breast cancer: meta-analyses of individual patient data from randomised trials. Lancet.

[15] Gnant M, Pfeiler G, Steger GG, Egle D, Greil R, Fitzal F (2019). Adjuvant denosumab in postmenopausal patients with hormone receptor-positive breast cancer (ABCSG-18): disease-free survival results from a randomised, double-blind, placebo-controlled, phase 3 trial. Lancet Oncol.

[16] Brown J, Rathbone E, Hinsley S, Gregory W, Gossiel F, Marshall H (2018). Associations between serum bone biomarkers in early breast cancer and development of bone metastasis: results from the AZURE (BIG01/04) trial. J Natl Cancer Inst.

[17] Tiidus PM (2011). Benefits of estrogen replacement for skeletal muscle mass and function in post-menopausal females: evidence from human and animal studies. Eurasian J Med.

[18] Dieli-Conwright CM, Spektor TM, Rice JC, Sattler FR, Schroeder ET (2012). Hormone therapy and maximal eccentric exercise alters myostatin-related gene expression in postmenopausal women. J Strength Cond Res.

[19] Ballinger TJ, Reddy A, Althouse SK, Nelson EM, Miller KD, Sledge JS. Impact of primary breast cancer therapy on energetic capacity and body composition. Breast Cancer Res Treat. 2018. 10.1007/s10549-018-4924-6 PMC6208924 30136009

[20] Sheean P, Gomez-Perez S, Joyce C, O'Connor P, Bojko M, Smith A, et al. Myosteatosis at diagnosis is adversely associated with 2-year survival in women with estrogen receptor-negative metastatic breast cancer. Breast Cancer Res Treat. 2021. 10.1007/s10549-021-06358-6 34389926

[21] Caan BJ, Cespedes Feliciano EM, Prado CM, Alexeeff S, Kroenke CH, Bradshaw P (2018). Association of muscle and adiposity measured by computed tomography with survival in patients with nonmetastatic breast cancer. JAMA Oncol.

[22] Deluche E, Leobon S, Desport JC, Venat-Bouvet L, Usseglio J, Tubiana-Mathieu N (2018). Impact of body composition on outcome in patients with early breast cancer. Support Care Cancer.

[23] Shachar SS, Deal AM, Weinberg M, Nyrop KA, Williams GR, Nishijima TF (2017). Skeletal muscle measures as predictors of toxicity, hospitalization, and survival in patients with metastatic breast cancer receiving taxane-based chemotherapy. Clin Cancer Res.

[24] Rier HN, Jager A, Sleijfer S, van Rosmalen J, Kock M, Levin MD (2017). Low muscle attenuation is a prognostic factor for survival in metastatic breast cancer patients treated with first line palliative chemotherapy. Breast.

[25] Villasenor A, Ballard-Barbash R, Baumgartner K, Baumgartner R, Bernstein L, McTiernan A (2012). Prevalence and prognostic effect of sarcopenia in breast cancer survivors: the HEAL Study. J Cancer Surv.

[26] Prado CM, Baracos VE, McCargar LJ, Reiman T, Mourtzakis M, Tonkin K (2009). Sarcopenia as a determinant of chemotherapy toxicity and time to tumor progression in metastatic breast cancer patients receiving capecitabine treatment. Clin Cancer Res.

[27] Damrauer JS, Stadler ME, Acharyya S, Baldwin AS, Couch ME, Guttridge DC (2018). Chemotherapy-induced muscle wasting: association with NF-kappaB and cancer cachexia. Eur J Transl Myol.

[28] Bentzinger CF, Lin S, Romanino K, Castets P, Guridi M, Summermatter S (2013). Differential response of skeletal muscles to mTORC1 signaling during atrophy and hypertrophy. Skelet Muscle.

[29] Hain BA, Jude B, Xu H, Smuin DM, Fox EJ, Elfar JC, et al. Zoledronic acid improves muscle function in healthy mice treated with chemotherapy. J Bone Miner Res. 2019. 10.1002/jbmr.3890 31614017

[30] Shachar SS, Williams GR, Muss HB, Nishijima TF (2016). Prognostic value of sarcopenia in adults with solid tumours: a meta-analysis and systematic review. Eur J Cancer.

[31] Martin L, Birdsell L, Macdonald N, Reiman T, Clandinin MT, McCargar LJ (2013). Cancer cachexia in the age of obesity: skeletal muscle depletion is a powerful prognostic factor, independent of body mass index. J Clin Oncol.

[32] Williams GR, Deal AM, Muss HB, Weinberg MS, Sanoff HK, Nyrop KA (2017). Skeletal muscle measures and physical function in older adults with cancer: sarcopenia or myopenia?. Oncotarget.

[33] Hirvensalo M, Rantanen T, Heikkinen E (2000). Mobility difficulties and physical activity as predictors of mortality and loss of independence in the community-living older population. J Am Geriatr Soc.

[34] Ostir GV, Kuo YF, Berges IM, Markides KS, Ottenbacher KJ (2007). Measures of lower body function and risk of mortality over 7 years of follow-up. Am J Epidemiol.

[35] Sehl M, Lu X, Silliman R, Ganz PA (2013). Decline in physical functioning in first 2 years after breast cancer diagnosis predicts 10-year survival in older women. J Cancer Surviv.

[36] Locquet M, Beaudart C, Reginster JY, Petermans J, Gillain S, Quabron A (2017). Prevalence of Concomitant bone and muscle wasting in elderly women from the SarcoPhAge cohort: preliminary results. J Frailty Aging.

[37] Sjoblom S, Suuronen J, Rikkonen T, Honkanen R, Kroger H, Sirola J (2013). Relationship between postmenopausal osteoporosis and the components of clinical sarcopenia. Maturitas.

[38] Levinger I, Scott D, Nicholson GC, Stuart AL, Duque G, McCorquodale T (2014). Undercarboxylated osteocalcin, muscle strength and indices of bone health in older women. Bone.

[39] Schiaffino S, Mammucari C (2011). Regulation of skeletal muscle growth by the IGF1-Akt/PKB pathway: insights from genetic models. Skelet Muscle.

[40] Sartori R, Schirwis E, Blaauw B, Bortolanza S, Zhao J, Enzo E (2013). BMP signaling controls muscle mass. Nat Genet.

[41] Regan JN, Trivedi T, Guise TA, Waning DL (2017). The role of TGFbeta in bone-muscle crosstalk. Curr Osteoporos Rep.

[42] Waning DL, Mohammad KS, Reiken S, Xie W, Andersson DC, John S (2015). Excess TGF-beta mediates muscle weakness associated with bone metastases in mice. Nat Med.

[43] Santa-Maria CA, Bardia A, Blackford AL, Snyder C, Connolly RM, Fetting JH (2018). A phase II study evaluating the efficacy of zoledronic acid in prevention of aromatase inhibitor-associated musculoskeletal symptoms: the ZAP trial. Breast Cancer Res Treat.

[44] Dieli-Conwright CM, Courneya KS, Demark-Wahnefried W, Sami N, Lee K, Sweeney FC (2018). Aerobic and resistance exercise improves physical fitness, bone health, and quality of life in overweight and obese breast cancer survivors: a randomized controlled trial. Breast Cancer Res.

[45] Thompson WR, Rubin CT, Rubin J (2012). Mechanical regulation of signaling pathways in bone. Gene.

[46] Uzer G, Fuchs RK, Rubin J, Thompson WR (2016). Concise review: plasma and nuclear membranes convey mechanical information to regulate mesenchymal stem cell lineage. Stem Cells.

[47] Sen B, Xie Z, Case N, Thompson WR, Uzer G, Styner M (2014). mTORC2 regulates mechanically induced cytoskeletal reorganization and lineage selection in marrow-derived mesenchymal stem cells. J Bone Miner Res.

[48] Case N, Thomas J, Xie Z, Sen B, Styner M, Rowe D (2013). Mechanical input restrains PPARgamma2 expression and action to preserve mesenchymal stem cell multipotentiality. Bone.

[49] Rubin C, Xu G, Judex S (2001). The anabolic activity of bone tissue, suppressed by disuse, is normalized by brief exposure to extremely low-magnitude mechanical stimuli. FASEB J.

[50] Qin YX, Rubin CT, McLeod KJ (1998). Nonlinear dependence of loading intensity and cycle number in the maintenance of bone mass and morphology. J Orthop Res.

[51] Frechette DM, Krishnamoorthy D, Adler BJ, Chan ME, Rubin CT (2015). Diminished satellite cells and elevated adipogenic gene expression in muscle as caused by ovariectomy are averted by low-magnitude mechanical signals. J Appl Physiol.

[52] Krishnamoorthy D, Frechette DM, Adler BJ, Green DE, Chan ME, Rubin CT (2016). Marrow adipogenesis and bone loss that parallels estrogen deficiency is slowed by low-intensity mechanical signals. Osteoporos Int.

[53] Gilsanz V, Wren TA, Sanchez M, Dorey F, Judex S, Rubin C (2006). Low-level, high-frequency mechanical signals enhance musculoskeletal development of young women with low BMD. J Bone Miner Res.

[54] Dutra MC, de Oliveira ML, Marin RV, Kleine HC, Silva OL, Lazaretti-Castro M (2016). Whole-body vibration improves neuromuscular parameters and functional capacity in osteopenic postmenopausal women. Menopause.

[55] Mogil RJ, Kaste SC, Ferry RJ, Hudson MM, Mulrooney DA, Howell CR (2016). Effect of low-magnitude, high-frequency mechanical stimulation on BMD among young childhood cancer survivors: a randomized clinical trial. JAMA Oncol.

[56] Pagnotti GM, Pattyn, R., Wright L.E., et al.: Mechanical signals preserve bone and muscle while suppressing adiposity in a murine model of complete estrogen deprivation. Presented at: American Society of Bone and Mineral Research Annual Meeting 2018.

[57] Pagnotti GM, Adler BJ, Green DE, Chan ME, Frechette DM, Shroyer KR (2012). Low magnitude mechanical signals mitigate osteopenia without compromising longevity in an aged murine model of spontaneous granulosa cell ovarian cancer. Bone.

[58] Pagnotti GM, Chan ME, Adler BJ, Shroyer KR, Rubin J, Bain SD (2016). Low intensity vibration mitigates tumor progression and protects bone quantity and quality in a murine model of myeloma. Bone.

[59] Yi X WL, Pagnotti GM, Uzer G, Powell KM, Wallace J, Sankar U, Rubin CT, Mohammad K, Guise TA, Thompson WR. Mechanical suppression of breast cancer cell invasion and paracrine signaling requires nucleo-cytoskeletal connectivity. 2019;bioRxiv 838359. 10.1038/s41413-020-00111-3 PMC7673025 33298883

[60] Matsumoto A, Hieda M, Yokoyama Y, Nishioka Y, Yoshidome K, Tsujimoto M (2015). Global loss of a nuclear lamina component, lamin A/C, and LINC complex components SUN1, SUN2, and nesprin-2 in breast cancer. Cancer Med.

